# Mediterranean Diet, Microbiota and Immunity

**DOI:** 10.3390/nu14020273

**Published:** 2022-01-10

**Authors:** Francisco J. Pérez-Cano

**Affiliations:** 1 Department of Biochemistry and Physiology, Faculty of Pharmacy and Food Science, University of Barcelona, 08028 Barcelona, Spain; franciscoperez@ub.edu; Tel.: +34-9340-24505; 2 Nutrition and Food Safety Research Institute (INSA-UB), 08921 Santa Coloma de Gramenet, Spain

It is well established that the diet, among other external influencing factors, also known as the exposome, has a key role in the prevention and management of different diseases. The health benefits of dietary patterns are derived from the nutritional and non-nutritional bioactive compounds (BC) present, which exert a direct effect on the individual (firstly on the intestinal epithelial barrier and then on distant tissues), on the microbiota (composition and functionality), and on the immune system (the intestinal immunity and also the systemic). Of the different occidental diets, the Mediterranean diet (MD) is the one with more scientific evidence suggesting a positive impact on health and describing the mechanisms involved. So far, in the last 5 years (2017–2021), a search on scientific platforms leads to more than five thousand publications for “Mediterranean Diet AND Health”, whereas a search focused on the overall mechanisms involved, such as “Mediterranean Diet AND Microbiota” or “Mediterranean Diet AND Immunity”, only accounts for around 250 publications each.

The MD is a plant-based eating pattern that was typical of countries such as Greece, Spain, and Italy before the pre-globalized food system, which is associated with better health due to the habitual eating of cereals, legumes, vegetables, fruits, olive oil and nuts, among others. These foods provide fiber with prebiotic activity, polyunsaturated fatty acids with anti-inflammatory properties, a variety of BC with antioxidative properties such as polyphenols, and vitamins and minerals in appropriate amounts. The adequate balance of these components leads to the proper modulation of the microbiota and the activation of the host (immunity) responses involved in health promotion and disease prevention. In this regard, two interesting review articles were published in *Nutrients* this year: the first one focused on the MD interplay with the gut microbiota and immunity [1] and the second one describing the mechanisms involved in these effects by flavanols, a particular subset of flavonoids [2].

García-Montero and colleagues [1] first described the structure, diversity and dynamics of the gut microbiota involved in the eubiosis and dysbiosis present in health and disease, respectively. Later, and after reviewing the intestinal immunobiology, they elegantly connected both systems and went deeper into the mechanisms involved, focusing on the receptors and pathways activated by the different microbial communities and their products. Furthermore, they highlighted the activation of the pattern recognition receptors (PRRs), the balance between T cell populations (Th17 vs. Treg), and the precise interaction of critical bacterial metabolites, such as short-chain fatty acids (SCFA) with host receptors. Later, and after introducing the MD main components, they accurately compiled the role of dietary components (monounsaturated and polyunsaturated fatty acids, fruits and vegetables rich in polyphenols, dietary fiber, vitamins and trace elements) abundant in MD, on the gut microbiota, immune system, and intestinal barrier, and the impact at systemic level. Finally, the authors addressed the impact of the Western diet as a model of unhealthy eating, in terms of the frequent intake of refined carbohydrates, unhealthy fats, excessive meat and fast-food consumption, salt and additives. 

As previously mentioned, the MD is very rich in polyphenols, which are BC from the secondary metabolism of plants present in fruits and vegetables that exert a wide range of beneficial effects. Polyphenols are divided into four principal classes: phenolic acids, flavonoids, stilbenes, and lignans. Martín and Ramos [2] focused their review on a specific subset of flavonoids, the flavanols. Particularly, and after summarizing the interplay of gut microbiota, immunity and inflammation, they centered their article on the impact of flavanols on the gut microbiota and metabolic diseases. A summary of the most relevant mechanisms of flavanol and flavanol-rich products involved in immunity, inflammation, and metabolism in the context of diabetes, obesity, and metabolic syndrome, either in cultured cells or in animal and human studies, was included. Moreover, the role of the microbiota in the above situations was also addressed. 

Overall, both articles provided an overview of the mechanisms involved in the modulation of the microbiota and the immune system by diet. In brief, many non-digestible dietary components, such as polyphenols or fiber, can act as prebiotics or can modulate microbiota or host functionality after microbiota metabolism (microbiota-dependent mechanisms). In addition, other microbial-independent mechanisms from the nutritional compounds induce host benefits, such as direct interaction with receptors from the epithelial barrier or the intestinal immune cells. Furthermore, direct anti-infective properties of diet components have been proposed [3,4].

Since the publication of these reviews in early 2021, the evidence has continued to grow, as well as the knowledge about the possible mechanisms involved in disease, and also at different stages of life. On the one hand, and regarding metabolism, the MD is linked to specific functional and taxonomic components of the gut microbiome that are positively associated with protective cardiometabolic health [5]. On the other hand, others have focused on the role of MD on age-related disease risk and longevity, not only showing its positive contribution but also describing many other mechanisms used by the dietary components of this healthy eating pattern [6]. Shannon et al. describe the importance of these components in protecting against genomic instability by preventing DNA damage, enhancing DNA repair, or attenuating telomere shortening, among others [6]. Finally, the microbiota composition and the components of the diet during pregnancy also seem to have a role in the immune status of both the mother and the infant at birth, especially when the plasmatic levels of cytokines and immunoglobulins are studied, the results being more relevant in the baby [7]. Overall, the diet–microbiota–immunity triad is a fascinating network that deserves to be studied globally, focusing on the dietary-specific effects and mechanisms, and also in different contexts, both in disease and health (early or later in life), and even in the programming process.

In summary, strong evidence exists for the multidirectional interaction of the dietary components with the microbiota and immunity, indicating how all three systems connect their pathways, influencing each other’s actions. Thus, the analysis of diet and microbiota composition should receive more attention when studying the immune or inflammatory state of a particular subject. Among the possible interventions to modulate this complex interplay, it seems that the MD is a very good option.

## References

[1] García-Montero C., Fraile-Martínez O., Gómez-Lahoz A.M., Pekarek L., Castellanos A.J., Noguerales-Fraguas F., Coca S., Guijarro L.G., García-Honduvilla N., Asúnsolo A. (2021). Nutritional Components in Western Diet Versus Mediterranean Diet at the Gut Microbiota-Immune System Interplay. Implications for Health and Disease. Nutrients.

[2] Martín M.Á., Ramos S. (2021). Impact of Dietary Flavanols on Microbiota, Immunity and Inflammation in Metabolic Diseases. Nutrients.

[3] Azagra-Boronat I., Rodríguez-Lagunas M.J., Castell M., Pérez-Cano F.J., Watson R.R., Preedy V.R. (2019). Prebiotics for gastrointestinal infections and acute diarrhea. Dietary Interventions in Gastrointestinal Diseases.

[4] Pérez-Cano F.J., Massot-Cladera M., Rodríguez-Lagunas M.J., Castell M. (2014). Flavonoids Affect Host-Microbiota Crosstalk through TLR Modulation. Antioxidants.

[5] Wang D.D., Nguyen L.H., Li Y., Yan Y., Ma W., Rinott E., Ivey K.L., Shai I., Willett W.C., Hu F.B. (2021). The gut microbiome modulates the protective association between a Mediterranean diet and cardiometabolic disease risk. Nat. Med..

[6] Shannon O.M., Ashor A.W., Scialo F., Saretzki G., Martin-Ruiz C., Lara J., Matu J., Griffiths A., Robinson N., Lillà L. (2021). Mediterranean diet and the hallmarks of ageing. Eur. J. Clin. Nutr..

[7] Rio-Aige K., Azagra-Boronat I., Massot-Cladera M., Selma-Royo M., Parra-Llorca A., González S., García-Mantrana I., Castell M., Rodríguez-Lagunas M.J., Collado M.C. (2021). Association of Maternal Microbiota and Diet in Cord Blood Cytokine and Immunoglobulin Profiles. Int. J. Mol. Sci..

